# C**urrent Strategies for Noble Metal Nanoparticle Synthesis**

**DOI:** 10.1186/s11671-021-03480-8

**Published:** 2021-03-15

**Authors:** Giyaullah Habibullah, Jitka Viktorova, Tomas Ruml

**Affiliations:** Department of Biochemistry and Microbiology, University of Chemistry and Technology Prague, Technická 5, 166 28 Prague, Czech Republic

**Keywords:** Noble metal nanoparticles, Personal healthcare, Therapeutics, Diagnostics, Biomedical applications

## Abstract

Noble metals have played an integral part in human history for centuries; however, their integration with recent advances in nanotechnology and material sciences have provided new research opportunities in both academia and industry, which has resulted in a new array of advanced applications, including medical ones. Noble metal nanoparticles (NMNPs) have been of great importance in the field of biomedicine over the past few decades due to their importance in personalized healthcare and diagnostics. In particular, platinum, gold and silver nanoparticles have achieved the most dominant spot in the list, thanks to a very diverse range of industrial applications, including biomedical ones such as antimicrobial and antiviral agents, diagnostics, drug carriers and imaging probes. In particular, their superior resistance to extreme conditions of corrosion and oxidation is highly appreciated. Notably, in the past two decades there has been a tremendous advancement in the development of new strategies of more cost-effective and robust NMNP synthesis methods that provide materials with highly tunable physicochemical, optical and thermal properties, and biochemical functionalities. As a result, new advanced hybrid NMNPs with polymer, graphene, carbon nanotubes, quantum dots and core–shell systems have been developed with even more enhanced physicochemical characteristics that has led to exceptional diagnostic and therapeutic applications. In this review, we aim to summarize current advances in the synthesis of NMNPs (Au, Ag and Pt).

## Introduction

Noble metals have been in use for a very long time, dating back to the first Egyptian civilization, and have always been viewed as a sign of superior power and wealth. As a result, they can be seen in history in the form of expensive artworks, coins, jewels, etc. [1]. These metals generally tend to be more expensive than others because of their availability in the Earth’s crust [2, 3]. Due to their robust nature, resistance to extreme conditions of corrosion and oxidation, they have been widely used in the aerospace, automotive, chemical, energy, electrical and electronics industry and more importantly healthcare (from surgical equipment to contrast enhancers in imaging) [4, 5].

Over the past two decades, nanotechnology has proven to be the most promising future technology, offering countless possibilities. Multidisciplinary support from academic and industrial sectors has made it the most rapidly expanding field, with highly promising outcomes [6–8]. Currently, the technological leap in synthesizing and controlling metals at the nanoscale level has provided immense research opportunities to progress in personalized healthcare, diagnostics and therapies [9–11]. Metal nanoparticles (MNPs) have turned out to be the most commonly and broadly studied because of their impressive physicochemical properties and large surface-to-volume ratio compared to their bulk material (metal). As for biomedical applications, NMNPs became a natural pick due to their resistance to harsh environments. They have been applied in highly sensitive diagnostic assays, as thermal ablation enhancers in radiotherapy, and as drug and gene delivery vehicles [3, 12, 13].

The recent merging of nanotechnology with material sciences has resulted in the development of new nanocomposite materials with highly enhanced thermal, catalytic, electrical, optical and mechanical properties compared to the individual components. Notably, composites made of NMNPs have gained a great deal of research interest because of their impressive physicochemical properties that play a vital role in modifying the nanoscale building blocks and result in wide applications in catalysis (mainly electrocatalysis), optics, nanomedicine and environmental protection [14–17]. Noble metals in the colloidal state have been the subject of intensive studies, mainly due to their effectiveness in therapeutics and diagnostics [2, 18]. Similarly, improvements in the synthesis of materials such as graphene oxide and reduced graphene oxide [14, 19, 20], quantum dots [21–23] and carbon nanotubes [24–26] has contributed to more feasible and effective methods for the formation of NMNCs.

Due to the small size of Au and PtNPs and NMNPs, their large surface area-to-volume ratio and ability to assist in high electron transfer processes, they are ideal candidates for applications as electrochemical sensors [27–29]. The optical properties of NMNPs have served as a topic for many studies, especially Ag and AuNPs. These NPs are able to respond differently to different wavelengths of light (extensive scattering from the visible region to the near infrared region with Au), and so they are applied as signal enhancers in surface-enhanced Raman spectroscopy (SERS), localized surface plasmon resonance and other resonance scattering spectroscopy [30–33]. Due to the extensively tunable optical properties and biocompatibility of AuNPs, they have been applied in the photothermal therapy and in vivo imaging (photoacoustic imaging) of tumors [34–36]. Recently, AgNPs have also exhibited their potential in photothermal therapy, where they are generally applied as Ag core–shell systems or composites (with reduced graphene oxide/ carbon nanotubes) [37–39]. The biocompatibility of NMNPs with cells and tissues has opened up broad applicability in diagnostics [14]. Biosensors of NMNPs and NMNCs (especially graphene) have played a key role in enhancements of accuracy and specificity that provide an advantage over existing biomolecular diagnostics methods [40, 41]. Generally, Au and PtNPs are employed in the development of novel biosensors and probes due to their ability to adsorb to the biomolecules along with their supreme conductivity and stability [42–45]. As a result, NMNPs themselves or in the form of NMNCs are applied as immunosensors [46], biomolecules for detection [47] and nanoprobes (for in vivo cell imaging, tracking and studying the pathogenesis of disease progression) [2, 6, 48]. Despite all these advantages of NMNPs and NMNCs, there have still been many questions and debates concerning their safety profile in the human body [49–51].

In this review, we provide a survey on the synthesis methodologies of NMNPs (Ag, Au and Pt) and NMNCs (with Ag, Au and Pt) along with their current developments in biomedical applications as therapeutics and diagnostics, including the synergism exhibited by NMNCs with NMNPs in terms of improved performance, which is a current hot topic in materials research.

## Current Trends in NMNPs Synthesis

### Synthesis Methods of NMNPs

**Fig. 1:**
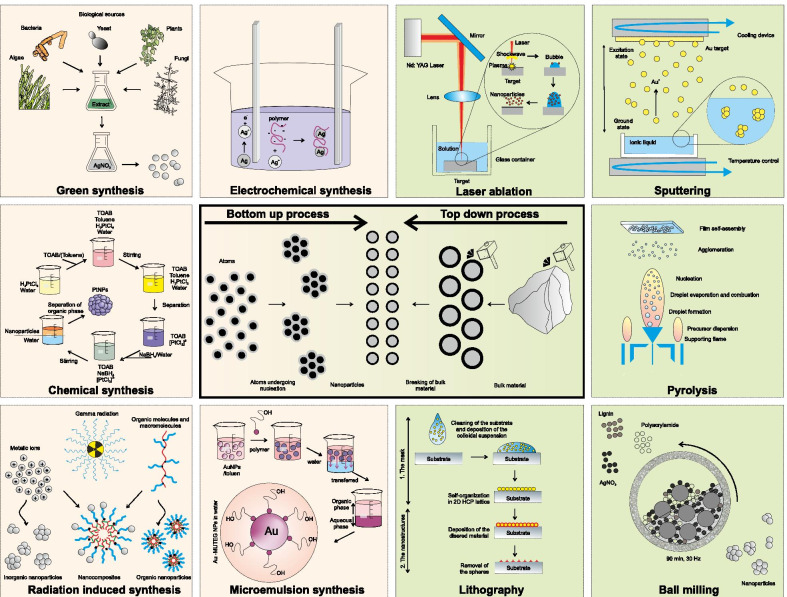
Schematic representation of the top-down (images with the green background) and bottom-up (images with pale yellow background) approaches of nanoparticle synthesis, the image was adapted and redrawn from [52–60]

The preparation of NPs basically follows two different approaches, (1) top-down (destructive method) and (2) bottom-up (constructive method) (Fig. 1).

Top-down processes involve breaking bulk materials into smaller particles of nano-dimensions using various physical and chemical methods. In contrast, in the bottom-up approach, NPs are produced by the self-assembly of the atoms, the molecules or the clusters. Top-down approaches involve externally controlled processes of cutting, milling and shaping the materials into the desired order and shape. Several physical methods, such as pyrolysis [61, 62], nanolithography [63, 64], thermolysis [65] and radiation-induced methods [66–68] belong in this category. However, this approach comes with a major limitation, which is the imperfect surface structure of the resulting MNPs, which substantially affects their physical and chemical properties [1]. Moreover, this method requires an enormous amount of energy to maintain the high-pressure and high-temperature conditions during the synthetic procedure, making the process expensive.

In bottom-up methods, NPs are assembled from the corresponding atoms, clusters and molecules using chemical as well as biological procedures. The bottom-up approach has turned out to be advantageous, as it provides a far better control over the final product formation with more homogeneous size, shape (physical parameters) and chemical composition. Moreover, this approach in general is less expensive. The bottom-up approach is commonly a wet-chemical synthesis procedure, such as chemical [69, 70], electrochemical [71–73], sonochemical [74, 75] and green synthesis [76, 77]. In the bottom-up approach, the purification of the synthesized particles from their reaction mixture (toxic chemicals, organic solvents and reagents) is a major challenge that casts doubt on their biomedical applications except for green synthesis methods.

### Top-Down Approaches

#### Sputtering

Sputtering is one of the most commonly used synthesis protocols that includes the deposition of NPs as a thin layer generated by the collision of ions over the substrate and followed by annealing. This method is also referred as the physical vapor deposition (PVD) method [78, 79]. The efficiency of this method mainly depends on factors such as layer thickness, substrate type, annealing duration and temperature, which directly influence the size and shape of the NPs [55, 80, 81].

#### Micropatterning

Micropatterning, a popular technique employed in biosensors, microarrays, tissue engineering and cellular studies [82], is also used in the synthesis of MNPs. In general, this technique is equivalent to a printing process in which a material is cut or formed into the required shape and size either with a light or electron beam for the synthesis of nanostructured arrays from an appropriate precursor. This is a low-temperature, non-vacuum method that uses photolithography for the synthesis of MNPs, employing the laser sintering of MNP ink [83, 84]. Apart from photolithography, numerous lithography techniques have been developed such as scanning, soft nanoimprinting, colloidal, nanosphere and E-beam lithography [2, 57, 85, 86].

#### Milling

Milling is generally represented as the public face of top-down processes, as it involves the direct breaking of bulk materials into micro/nanostructures. In mechanical milling, the kinetic energy of the rollers/balls is transferred to the bulk material, which results in the reduction in grain size [87]. Parameters such as the type of mill, milling atmosphere, milling media, intensity, time and temperature play a crucial role in controlling the shape and size of the NPs [88, 89]. Different techniques have been developed in order to overcome these constraints, including shaker mills, tumbler mills, vibratory mills, attrition mills and planetary mills.

#### Laser Ablation

Laser ablation is one of the methods that is considered to be a suitable replacement for conventional chemical methods due to its fast processing times, providing better control over the size and shape of the particles and high yields with better long-term stability [78, 90–92]. In a laser ablation process, a solid surface (generally a plate of pure metal) is irradiated with a laser beam, leading to a low-flux plasma plume, which is finally evaporated or sublimated to form NPs [93]. At a higher flux, the materials are converted to plasma. The lack of requirement to remove excess reagents as well as the possibility of metal nanoparticle synthesis in both aqueous and organic solvents has enabled the implementation of the laser ablation method in biomedical applications such as the in situ conjugation of biomolecules with MNPs, which has been proved to be more effective than standard techniques [54, 94, 95].

#### Pyrolysis

Thermal decomposition is another important technique commonly used separately or in combination with other physical methods for MNP synthesis [78]. It is an endothermic chemical decomposition process that uses heat to break the compound’s chemical bonds, resulting in decomposition of the precursor, forcing it into a chemical reaction producing NPs along with other by-products in the form of ash. Through further processing of the obtained solid ash, NPs are recovered. Pyrolysis is frequently used for the preparation of noble MNPs [56, 96, 97]. Excessive energy consumption is one of the most important drawbacks of this method.

#### Chemical Vapor Deposition

This method is also known as the vacuum deposition method, where the gaseous reactant is deposited as a thin film onto a substrate along with a combination of other gas molecules that promote superheating of the substrate. During the reaction, the substrate comes in contact with the combined gases, leading to reduction of the ions [78]. The product of this reaction is usually in the form of a film which the NPs need to be scraped out from. The method produces highly pure, uniform and nonporous nanoparticles; as a result, this method has become highly important in the electronics and semiconductor industry. Despite these huge advantages, this method suffers from some major disadvantages: The requirement for special equipment for making the films and chambers for the reaction, and the fact that the gaseous by-products of this reaction are extremely toxic [98].

### Bottom-Up Approaches

#### Reduction of Metal Ions in Solution

This approach involves the reduction of metal ions from their ionic salts by using various chemical reducing agents in the presence of a stabilizing agent under favorable reaction parameters (pH, temperature, etc.). This procedure is the most common and reliable method of all the bottom-up approaches due to its sheer simplicity [2, 99]. An extensive list of a number of reducing agents is available for this process that includes commonly used sodium citrate [10, 100], tannic acid [99], sodium borohydrate [101], hydrazine, hydrogen, lithium aluminum hydride, and alcohols can also be used [2, 60]. Similarly, when it comes to stabilizing agents there are many options, and they generally fall into two categories (1) low-molecular-weight (e.g., citrate, SDS, chitosan, etc.) and (2) high-molecular-weight ones (e.g., starch, tween, PVP, PEG, DISPERBYK, etc.). The low-molecular-weight stabilizers (generally charged detergents) have the tendency to alter the surface charge of the synthesized particles and maintain the repulsive force between them, preventing aggregation; this type of stabilizer generally does not protect well against environmental stress factors (especially changes in storage temperature and light exposure). High-molecular-weight stabilizers generally engulf the particles and protect them from environmental stresses. They have been shown to be more efficient than the low-molecular-weight stabilizers. Despite their advantages, their biological applications and catalytic properties are questionable due to the thick layer of stabilizing agent over the particles that prevents their dissolution [102, 103]. In terms of homogeneity in particle size and shape, the clear winner is the chemical-based reduction. This is because reduction can be easily regulated by changing the reaction parameters (pH and the ratio between the reducing and the stabilizing agent). Tyagi and his team produced AuNPs [104] using the citrate reduction method at room temperature, at pH 3 with 2:1 and 5:1 molar ratios of citrate to AuCl_3_ of, yielding particles with an average size of 28 and 25 nm, respectively. At this pH, the reaction was much faster than at other pH values. They also showed that AuNPs of different shapes such as prisms, rods and spheres were formed at pH values ranging from 3 to 6 (with a 2:1 molar ratio of citrate to AuCl_3_). In another study by Agnihotri and coworkers [105], who applied a similar citrate reduction method for the synthesis of AgNPs, obtained particles with an average size of 5 nm at the highest concentration of sodium citrate (4.28 × 10^–3^ mol dm^−3^). Their size increased at elevated concentrations of citrate (to 100 nm at 1.77 × 10^–2^ mol dm^−3^). Another study by Hou et al. [106] described the synthesis of highly stable and monodispersed Pt nanoparticles in the form of hydrosols for electrocatalytic applications.

#### Microemulsion

The fabrication of metal NPs based on microemulsions is becoming a topic of great interest, and it has also emerged as an effective method that provides better control over the physical aspects of the synthesized nanoparticles such as size and shape. In general, microemulsions are simply mixtures of two immiscible liquids in the presence of a surfactant. These systems generally have ultralow interfacial tension, a large interfacial area and thermodynamic stability [107]. The first microemulsion-based synthesis of NMNPs was described by the team of Muñoz-Flores et al. [58, 108, 109] who synthesized platinum, palladium and rhodium NPs. In the microemulsion-based NPs synthesis, two separate microemulsions are prepared, one containing the ionic salt and another containing the reducing agent produced in an amphiphilic environment. The collision between the emulsions leads to the mixing of the reactants and reduces the ions from the salt to neutral atoms, which then form nanoparticles [2]. Water-in-oil systems are generally employed for the synthesis of metal nanoparticles, and as the nanoparticles produced by this method are derived in the form of emulsions, they are generally thermodynamically stable. Depending on the need, this process could be also tailored to synthesize a specific type of nanoparticle by altering the ratio of the surfactant to oil. This makes it possible enables to control the size and shape of the particles [110].

#### Electrochemical Methods

Electrochemical processes are commonly employed for the synthesis of NMNPs and nanocomposites, which are mostly used for their catalytic properties and have recently been used in biomedical applications as biosensors [111]. The electrochemical method was first introduced in 1994 by Reetz and Helbig, who dissolved a pure metal sheet from the anode to achieve the deposition of metal salt on the cathode of an electrochemical cell in the presence of an electrolyte to produce nanoparticles [2, 112]. The effectiveness of this method depends on various parameters such as the nature of the reducing agent, the purity of the metal and the stabilizer, choice of the electrolyte, concentration ratio and temperature, which directly impact the physical parameters of the NPs [53]. At present, the synthesis of nanocomposites (especially those with graphene) using electrochemical methods is preferred to the synthesis of NPs [113].

#### Radiation-Induced Synthesis Methods

This method employs ionizing radiation (especially gamma radiation and includes X-rays and UV-light) for the synthesis of metal nanoparticles. It has been proved to be highly efficient compared to the conventional methods of NP synthesis, as it provides fully reduced, highly pure (by-product free) metal nanoparticles. The topic has been nicely covered in several reviews [59, 66, 114, 115]. In this process, an aqueous solution of reducing and stabilizing agent is exposed to radiation-mediated radiolysis, which leads to the formation of NPs. During the radiation exposure, the water molecules break up, yielding transient products that act as strong oxidizing or reducing agents and reduce metal ions to neutral metal atoms, which further nucleate to form NPs. The synchrotron X-ray techniques enabled monitoring of the growth trajectories of colloidal NPs in real time [116]. The physical parameters critical for the synthesis of NPs include the radiation dose, pH of the system and the type of solvent used in the synthesis [117]. Recently, radiation-induced synthesis was used for the production of tween 80 stabilized AgNPs for antibacterial applications [118].

#### Microwave-Induced Green Synthesis Methods

Generally, microwave-assisted synthesis is also known as one-pot synthesis and involves the synthesis of NPs from salts and surfactant solutions. It is a highly reliable, fast and easy method that supports control over the morphology of the synthesized NPs [2]. This method works on the principle of dipole interaction (molecules tend to align themselves and oscillate in step with the oscillating electrical field of the microwaves, collision and friction between them causes heat) and ionic conduction (The electric field generates ionic motion as the molecules try to orient themselves to the rapidly changing field, causing instantaneous super heating) producing a heating effect that results in the reduction of metal ions to NPs [119, 120]. The microwave irradiation time and the concentration of the reactant mainly determine the morphological parameters of the NPs. Recently, physical properties such as monodispersity and grain size of superparamagnetic magnetite NPs prepared by microwave-assisted synthesis were controlled by the injection of humate-polyanion at different stages of the synthesis [121]. Microwave-induced electric discharge was used also for the synthesis of Cu, Ni, und Zn nanoparticles from metal particles in the absence of solvents or surfactants [122].

#### Green Synthesis Methods

**Fig. 2:**
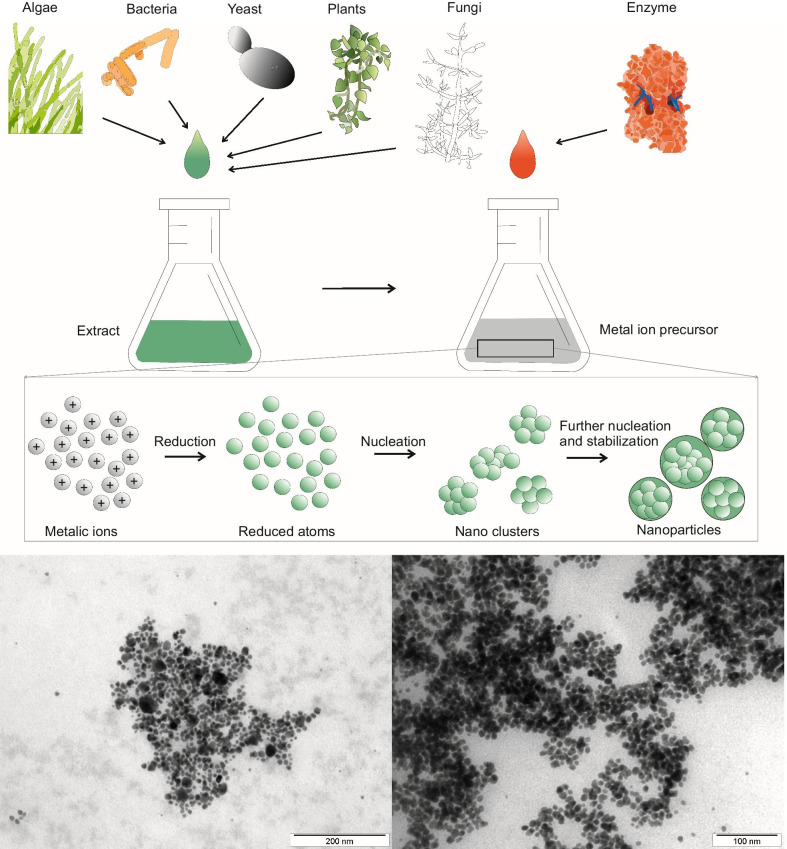
Schematic representation of green synthesis methods

The excessive use of chemicals in chemical synthesis has almost jeopardized the future of biological applications of NMNPs. This resulted in the exploration of other, ecological methods with a minimal use of chemicals. Green synthetic methods employing plant extracts, microorganisms and biopolymers have proven to be potent candidates for replacing chemical methods of NP synthesis (Fig. 2) [123]. Thanks to simpler and greener methodologies, there has been an exponential increase in publications in the past two decades [52, 124, 125].

#### Biosystem Synthesis of NMNPS

The quest for the development of economically and environmentally benevolent methods has led to the exploration of microorganisms as a potential candidate for the synthesis of nanoparticles [126, 127]. Biological systems are excellent examples of hierarchical organizations of atoms and molecules, which attract researchers to use microorganisms as potential cell factories for nanomaterial preparation. Both prokaryotic (bacteria) and eukaryotic (algae, fungi and plants) species are used for the green synthesis of NPs [123].

##### Bacteria-Based Synthesis of Nanoparticles

Bacteria that have been repeatedly exposed to metal-rich environments have often developed resistance to these extreme conditions [128]. Thus, prokaryotes have become a natural choice for producing nanomaterials. *Pseudomonas stutzeri* AG259, a metal-accumulating bacterium isolated from a silver mine, was utilized by Klaus et al. [129] to create intracellular nanocrystals of metallic silver of up to 200 nm in size. The extracellular synthesis of NPs was first reported by Shahverdi and co-workers [130], where AgNPs were produced by the reduction of aqueous Ag^+^ ions through various culture supernatants of Gram-negative bacteria, i.e., *Enterobacter cloacae*, *Escherichia coli* and *Klebsiella pneumonia*. The synthesis rate was much faster than the intracellular synthesis, which resulted in Ag-NPs synthesis within 5 min of the Ag + ions encountering the cell filtrate. Extracellular reductase enzymes produced by the microorganisms, namely *Bacillus licheniformis* and *Bacillus clausii*, reduce the silver ions to neutral silver, resulting in nanosized particles. Protein assay of these microorganisms revealed that the NADH-dependent reductase enzyme plays a vital role in the bioreduction of silver ions to silver nanoparticles. The reductase enzyme gets its electrons from NADH oxidation to NAD + . During the oxidation, the enzyme also gets oxidized at the same time, resulting in the reduction of silver ions to AgNPs. In some cases, it has been observed that the nitrate-dependent reductase can also participate in the bio reduction [131–133]. In addition, several bacterial strains (gram-negative as well as gram-positive), namely *A. calcoaceticus, B. amyloliquefaciens, B. flexus, B. megaterium* and *S. aureus*, have also been used for both the extra- and intracellular biosynthesis of AgNPs [123]. Similarly, AuNPs and PtNPs are also prepared by the accumulation and reduction of gold and platinum salts by bacteria. *B. licheniformis, B. megaterium*, *Delftia sp* KCM-006., *Shewanella sp*., *Stenotrophomonas maltophilia* and *Lactobacillus sp*. are some examples of bacteria which have been used to produce gold nanomaterials [134, 135]. In addition, the bacteria *Shewanella sp*. and *Acinetobacter calcoaceticus* PUCM 1011 were utilized for the preparation of PtNPs [136, 137]. Although bacteria-mediated synthesis is promising in terms of its green nature and control over the particle shape and size (mostly in extracellular synthesis), it suffers from disadvantages such as handling difficulties and low yields.

##### Fungus-Based Synthesis

In recent years, NMNP synthesis with eukaryotic microorganisms has emerged as a better alternative to prokaryotes due to their high intracellular metal uptake capability, ability to synthesize NPs with different chemical compositions, ability to produce a large amount of enzymes per unit biomass and easy biomass handling at laboratory scale [131].

In general, fungi have the potential to synthesize metallic NPs due to their metal bioaccumulation capacity, their tolerance, high binding capacity and intracellular uptake like bacteria [127]. Fungi use both intracellular and extracellular methods for the synthesis of NPs, and extracellular synthesis is the most commonly reported synthesis mechanism due to their ability to produce large quantity of extracellular enzymes that convert Ag^+^ ions to nanoscale silver particles [138–140]. In intracellular synthesis, Ag^+^ ions are adsorbed to the cell surface by the electrostatic interaction between negatively charged carboxylate groups in enzymes and positively charged Ag^+^ ions. Ag^+^ ions are later reduced by the enzymes present in the cell wall to form AgNPs, in this process NPs are formed on the surface of mycelia, not in solution. In 2001, the intracellular preparation of AuNPs using *Verticillium sp* was first reported by Mukherjee et al. [141], where Au^3+^ ions from tetrachloroaurate were reduced within the fungal cells, resulting in the formation of particles within the size range of 20 nm. Vahabi and coworkers [142] employed *Trichoderma reesei* for AgNPs synthesis, where the media with biomass was inoculated with AgNO_3_ and incubated over a period of 72 h, resulting in the formation of AgNPs in the size range of 5–50 nm. Similarly, another study by the team of Vigneshwaran et al. [138] demonstrated the intracellular synthesis of AgNPs from *Aspergillus flavus* and reported that enzymes in the cell wall were mainly responsible for the reduction, and the proteins were responsible for stabilization. Despite all these advantages such as faster synthesis, and better control over the size and shape of the synthesized particles, intracellular processes suffer from a huge disadvantage in terms of product recovery that makes the process hard and expensive, since NPs bind to the cell. As a result, extracellular synthesis is preferred. In extracellular synthesis, cell-free broth/suspension is used in the synthesis process that turns out to be more environmentally friendly and cost-effective. In 2016, the team of Balakumaran et al. [143] used a cell-free suspension of *Aspergillus terreus* for the synthesis of both Au and AgNPs, resulting in spherical nanoparticles in the size range of 8–20 nm and 10–50 nm for Ag and AuNPs, respectively. FTIR evaluation of the particles confirmed the binding of proteins with the NPs.

##### Algae-Based Synthesis

The algae-mediated synthesis of NPs utilizes four different methods: (1) whole algal cells are harvested from their culture media at a given phase of growth using centrifugation and then dispersed directly into an aqueous solution of the metallic salt; (2) cell-free aqueous extract made from freshly harvested or lyophilized cells; (3) an aqueous extract filtrate or supernatant of ground, fresh or dried algae; and (4) an aqueous filtrate of an algal broth. Extract-mediated synthesis is the most commonly reported algae-based synthesis mechanism [131, 144]. The accumulation of elemental gold in the form of AuNPs (9–20 nm) was noted with a dried cell suspension of *Chlorella vulgaris* by Hosea et al., who also reported an increase in the concentration of gold with time, proving the ability of the algal cells to uptake and reduce the gold ions from tetrachloroauric acid [145]. Velgosova and coworkers [146] reported on the synthesis of highly stable AgNPs from *Parachlorella kessleri*, a green algae aqueous extract, where the synthesized particles were in the size range of about 20 nm and exhibited excellent stability over a year. Other Algal sp, such as *Pithophora oedogonia*, *Sargassum wightii* and *Plectonema boryanum*, have been used successfully to construct Ag, Au and PtNPs, respectively [147–149].

##### Plant-Based Synthesis

Plant- and plant extract-mediated synthesis has been the most commonly reported synthesis methodology [123, 135, 150]. This type of synthesis is designated phytosynthesis. The major advantage of this synthesis method is easy product recovery. In 2003, the team of Gardea-Torresdey et al. was the first to illustrate the synthesis of metal nanoparticles (AgNPs) using a living plant system with alfalfa sprouts (*Medicago sativa*) in an agar medium. The roots possess the tendency to absorb the Ag from the medium and transport it along the shoot of the system in the same oxidation state, in the shoot the Ag atoms are further arranged to form AgNPs. Similarly, another study employed the alfalfa plant secretome to reduce Au^+^ to Au^0^, which also followed a similar procedure to produce AuNPs [151]. Plant-extract-mediated synthesis uses a plant component (leaves, stems, roots, shoots, flowers, barks and seeds) extract for the synthesis of NPs, the major advantage of this method is the ability of the extract to serve as both the reducing and stabilizing agent [152]. This method has been proved to be the most cost efficient and user friendly method to produce nanoparticles with long-term stability. In 2016, the team of Balashanmugam et al. demonstrated the phytogenic synthesis of AgNPs from *Cassia roxburghii* aqueous leaf extract. The synthesized AgNPs were in the size range of about 35 nm and exhibited excellent stability over a year. This method also facilitated the synthesis of both individual and bimetallic particles. Neem (*Azadirachta indica*) leaf extract was successfully used by Shankar et al. [153] to prepare silver, gold and bimetallic Au/Ag core–shell NPs. Similar plant extracts (bark, leaf, fruit and gum) have been used by several researchers to produce a variety of NMNPs [153–155]. Currently, light-induced nanoparticles are in the spotlight, as this procedure facilitates faster synthesis during the exposure of the mixture to sunlight. Kumar et al. [156] used *Erigeron Bonariensis* aqueous leaf extract for the synthesis of silver nanoparticles that yielded spherical and oval-shaped AgNPs with a size range of 13 nm (TEM size). The crucial parameters to be considered in this synthesis are the light exposure time and the concentration of the plant extract in the reaction system.

## Conclusion

Several physical, chemical as well as biological methods have been developed for the synthesis of NPs. All these processes are widely used based on the utility and applicability of the nanoproducts. However, each of the existing protocols suffers from certain drawbacks and also most of these processes cannot be scaled up for large-scale production. Thus, the development of alternative processes to fabricate NPs with controlled and tunable properties is still an open challenge.

## Data Availability

Not applicable.

## Abbreviations

NM: Noble metals
NPs: Nanoparticles
NMNPs: Noble metal nanoparticles
AuNPs: Gold nanoparticles
AgNPs: Silver nanoparticles
PtNPs: Platinum nanoparticles
NMNCs: Noble metal composites
PVD: Physical vapor deposition
SDS: Sodium dodecyl sulfate
PVP: Polyvinylpyrrolidon
PEG: Polyethylene glycol
AuCl_3_: Gold chloride
NADH: Nicotinamide adenine dinucleotide
TEM: Transmission electron microscopy
